# Lethal Respiratory Disease Associated with Human Rhinovirus C in Wild Chimpanzees, Uganda, 2013

**DOI:** 10.3201/eid2402.170778

**Published:** 2018-02

**Authors:** Erik J. Scully, Sarmi Basnet, Richard W. Wrangham, Martin N. Muller, Emily Otali, David Hyeroba, Kristine A. Grindle, Tressa E. Pappas, Melissa Emery Thompson, Zarin Machanda, Kelly E. Watters, Ann C. Palmenberg, James E. Gern, Tony L. Goldberg

**Affiliations:** Harvard University, Cambridge, Massachusetts, USA (E.J. Scully, R.W. Wrangham);; University of Wisconsin‒Madison, Madison, Wisconsin, USA (S. Basnet, K.A. Grindle, T.E. Pappas, K.E. Watters, A.C. Palmenberg, J.E. Gern, T.L. Goldberg);; University of New Mexico, Albuquerque, New Mexico, USA (M.N. Muller, M.E. Thompson);; Makerere University, Kampala, Uganda (E. Otali, D. Hyeroba);; Tufts University, Grafton, Massachusetts, USA (Z. Machanda)

**Keywords:** *Picornaviridae*, enterovirus, rhinovirus, rhinovirus C, human rhinovirus, chimpanzee, Uganda, respiratory disease, outbreak, epidemic, virology, epidemiology, asthma, cadherin, CDHR3, *CDHR3*-Y_529_, *CDHR3*-C_529_, viruses, common cold, respiratory infections, zoonoses, anthroponoses

## Abstract

We describe a lethal respiratory outbreak among wild chimpanzees in Uganda in 2013 for which molecular and epidemiologic analyses implicate human rhinovirus C as the cause. Postmortem samples from an infant chimpanzee yielded near-complete genome sequences throughout the respiratory tract; other pathogens were absent. Epidemiologic modeling estimated the basic reproductive number (*R*_0_) for the epidemic as 1.83, consistent with the common cold in humans. Genotyping of 41 chimpanzees and examination of 24 published chimpanzee genomes from subspecies across Africa showed universal homozygosity for the cadherin-related family member 3 *CDHR3*-Y_529_ allele, which increases risk for rhinovirus C infection and asthma in human children. These results indicate that chimpanzees exhibit a species-wide genetic susceptibility to rhinovirus C and that this virus, heretofore considered a uniquely human pathogen, can cross primate species barriers and threatens wild apes. We advocate engineering interventions and prevention strategies for rhinovirus infections for both humans and wild apes.

Rhinoviruses are antigenically diverse members of the family *Picornaviridae*, genus *Enterovirus*, that cause the common cold (*1*). Rhinovirus C causes an estimated 50% of all human upper respiratory tract infections (*1*) and most acute exacerbations of asthma in children (*2*). More than 160 distinct rhinovirus genotypes have been identified in human populations globally, and extensive antigenic heterogeneity limits cross-protective immunity (*3*). Accordingly, persons typically acquire several rhinovirus infections annually throughout childhood and continue to acquire new infections throughout adulthood (*4*).

Although many rhinovirus infections are only mildly symptomatic, rhinovirus C has been associated with influenza-like respiratory symptoms and acute exacerbations of asthma in children (*5*). The elevated virulence of rhinovirus C derives from its unique reliance on the cadherin-related family member 3 (CDHR3) receptor for host cell binding (*6*). A single-nucleotide polymorphism in *CDHR3* (rs6967330, C529Y) is associated with a 10-fold increase of RV-C binding and progeny yield and is a major risk factor for rhinovirus C infection and asthma (*7*). The protective *CDHR3*-C_529_ nonrisk allele has been detected exclusively in modern humans, whereas archaic Neanderthal and Denisovan humans each expressed the ancestral risk allele (*8*). The origin of rhinovirus C and the spread of the protective allele among modern human populations is thought to have occurred ≈8,000 years ago (*9*).

Humans and chimpanzees (*Pan troglodytes*) are closely related species that share many physiologic similarities, including a general predisposition for pathogen exchange, which, in some cases, might lead to human pandemics (*10*). Anthroponotic (i.e., of human origin) respiratory viruses have caused notable mortality rates in wild chimpanzee populations (*11*–*14*), and molecular testing of both postmortem and noninvasive (fecal) samples from wild apes during respiratory disease outbreaks have implicated human paramyxoviruses (family *Paramyxoviridae*) as anthroponotic causes (*11*–*14*). However, because of the limitations of field-based diagnostics, direct detection of pathogens from lesions of the respiratory tract has proven difficult (*12*,*14*). In this study, we were able to identify rhinovirus C, a pathogen not previously known to infect species other than humans, as the causative agent of an epidemic of respiratory disease in chimpanzees by directly sampling the lesions of a dead animal and by subsequent noninvasive sampling (from feces) and epidemiological modeling.

## Methods

### Ethics Statement

This study involved only observational, noninvasive research with wild chimpanzees. All animal protocols were approved by the Harvard University Institutional Animal Care and Use Committee (Cambridge, MA, USA; protocol 96-03) and the University of New Mexico Office of Animal Care Compliance (Albuquerque, NM, USA; protocol 11-100726-MCC). These protocols adhered to guidelines and laws set forth by the Weatherall Report on the use of nonhuman primates in research (https://royalsociety.org/policy/publications/2006/weatherall-report/), as well as the Guide for the Care and Use of Laboratory Animals of the National Institute of Health, Office of Animal Welfare, US Department of Agriculture Animal Welfare Act, Institute for Laboratory Animal Research Guide for the Care and Use of Laboratory Animals, US Public Health Service, US National Academies of Sciences National Research Council, and US Centers for Disease Control and Prevention.

### Sample Collection and Sequence Analysis

Starting approximately in February 2013 and continuing through August of that year, the Kanyawara community of chimpanzees in Kibale National Park, western Uganda, experienced an outbreak of severe respiratory disease. At the onset of the epidemic, the community consisted of 56 chimpanzees <1 week‒56.9 years of age. All Kanyawara chimpanzees are individually identifiable and habituated to human observers, such that trained field assistants collected direct data on behavior and respiratory signs daily. We compiled these data into weekly measures of clinical signs (coughing or sneezing, further classified as mild or severe) and deaths. During June‒August 2013, we collected fecal samples from 41 chimpanzees. Immediately after observing defecation by a chimpanzee, we placed 5 mL of the fecal sample into an equal volume of RNAlater buffer (Thermo Fisher Scientific, Waltham, MA, USA), homogenized the mixture, and stored the sample at −20°C for up to 3 months, until export to the United States.

One chimpanzee showing clinical signs (Betty, a 2.2-year-old female) died during the outbreak; her body was recovered immediately after death. An experienced veterinarian (D.H.) performed the postmortem examination and collected samples from her oropharynx, trachea, and lung using swabs (sterile, plastic shaft with Dacron tip) and stored the samples separately in 0.25 mL of RNAlater buffer at −20°C. We homogenized the swab tips, extracted sample RNA, and converted the RNA to double-stranded cDNA in the field. We shipped the cDNA to the United States for unbiased sequencing and pathogen discovery on an Illumina MiSeq instrument (Illumina, San Diego, CA, USA) using 300-bp paired-end read chemistry as previously described (*15*).

We analyzed sequence data using CLC Genomics Workbench version 8.5 (CLC bio, Aarhus, Denmark). In brief, we trimmed low-quality bases (phred quality score <30), discarded short reads (<75 bp), and subjected the remaining reads to de novo assembly. We then analyzed raw sequence reads and assembled contiguous sequences (contigs), which we examined for similarity to known viruses in GenBank databases.

From these analyses, we inferred the presence of a rhinovirus, which we subsequently identified as a member of the rhinovirus C species. We performed pairwise sequence comparisons in MEGA7 (*16*) to assess the genetic similarity of this virus to its relatives, and we evaluated recombination with other rhinovirus C strains by using RDP4 (*17*). We performed phylogenetic analyses on this virus and all complete rhinovirus polyprotein genes available in the GenBank database. We first aligned genes by codon using the MAFFT algorithm (*18*) implemented in Translator X (*19*) and removed poorly aligned regions using the Gblocks algorithm (*20*). We then used jModelTest (*21*) to estimate the model of molecular evolution from the data and PhyML (*22*) for phylogenetic inference.

We tested chimpanzee fecal samples for a suite of respiratory pathogens by multiplex Luminex assay using the NxTAG Respiratory Pathogen Panel (Luminex Corporation, Austin, TX, USA) (*23*) and confirmed positive results by singleplex PCR. The NxTAG Respiratory Pathogen Panel includes influenza virus A (multiple subtypes), human respiratory syncytial viruses A and B, coronaviruses (multiple subtypes), human metapneumovirus, rhinovirus/enterovirus, adenovirus, parainfluenza viruses 1–4, bocavirus, and the bacterial pathogens *Chlamydophila pneumoniae* and *Mycoplasma pneumoniae*. In brief, we added up to 50 µL of fecal suspension to 350 µL of saline; homogenized the mixture; and clarified the mixture by centrifugation at 15,000 rpm for 3 minutes, after which we added Luminex internal control MS2 before nucleic acid extraction with the NucliSENS EasyMag kit (bioMérieux, Marcy-l’Étoile, France). We then used 10 µL of eluate in the Luminex assay according to the manufacturer’s specifications. We applied this same assay to swab samples and assessed the viral load of rhinovirus C‒positive swab samples using a published real-time quantitative PCR (qPCR) (*24*).

### *CDHR3* Genotyping

Given the influence of *CDHR3* allelic variants on rhinovirus C pathogenesis in humans, we genotyped the *CDHR3* allele of 41 chimpanzees from the Kanyawara community using their fecal specimens. In brief, we extracted DNA from fecal samples using the MagMAX Kit (Thermo Fisher Scientific), and we then genotyped the extracted DNA using a qPCR-based allelic discrimination assay (CDHR3 TaqMan SNP Genotyping Assay; Thermo Fisher Scientific). We assayed serial 2-fold dilutions of eluted DNA to identify the dilution yielding the strongest fluorescent signal; then, we tested 2 additional replicates of each sample at that optimal dilution. We also examined the *CDHR3* locus of 24 additional chimpanzees from across Africa (including 4 recognized subspecies) using whole genome sequences previously published as part of the Great Ape Genome Project (*25*).

### Epidemiologic Modeling

To infer epidemiologic parameters related to transmission, we constructed an SIR (susceptible-infectious-removed) mathematical model. Following Althaus et al. (*26*), we fit the SIR model to cumulative incidence data and produced maximum-likelihood estimates of epidemiologic parameters (e.g., transmission rate, recovery rate) using the Nelder and Mead optimization algorithm in the optim package in R version 3.3.2 (https://www.r-project.org/). Because the epidemic was triphasic, we fit each of the three 2013 respiratory episodes separately. We assumed a well-mixed population of 50 chimpanzees and did not explore heterogeneity of social interactions to minimize model complexity and provide baseline estimates of epidemiologic parameters (Technical Appendix Figure 1).

**Figure 1:**
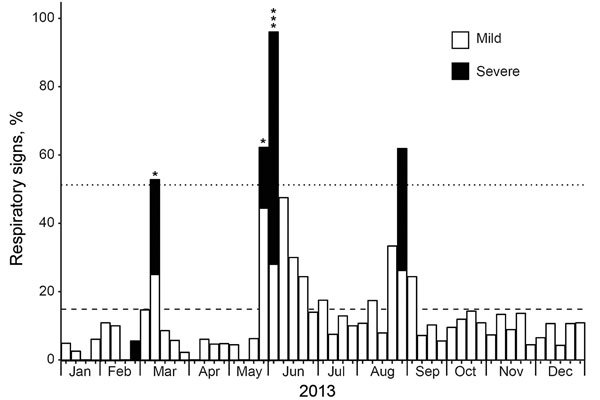
Epidemic curve of respiratory illness in the Kanyawara chimpanzee community, Uganda, 2013. Observational data on clinical severity (mild or severe) of respiratory signs (coughing and sneezing) were obtained and compiled into weekly measurements. The proportions of animals showing signs of respiratory illness are displayed by severity. Dashed line indicates 2013 mean rate of respiratory signs, and dotted line indicates 2 SD above that mean. Asterisks above bars indicate the timing of individual animal deaths.

## Results

The epidemic occurred in 3 phases (Figure 1): an early phase (21 days in February‒March 2013), during which 24 animals became ill and 1 infant died; a middle phase (22 days in May‒June 2013), during which 40 animals became ill and 4 adults died; and a late phase (19 days in August‒September 2013), during which 31 animals became ill and none died. Of the ≈56 chimpanzees in the community at the beginning of 2013, five died during the outbreak (1 infant 2.2 years of age and 4 adults 24.0‒57.9 years of age), for an overall mortality rate of 8.9%.

**Figure 2:**
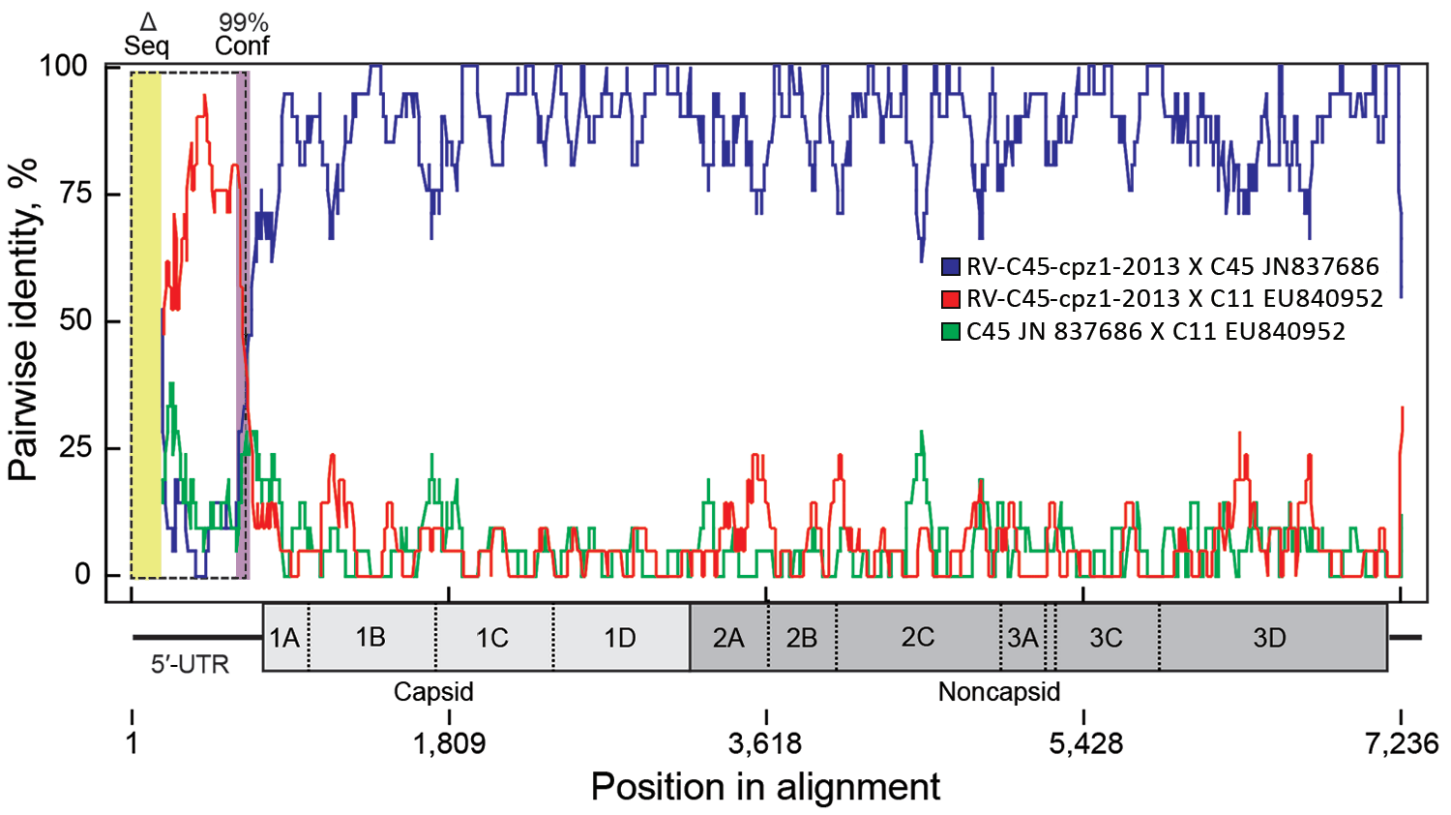
Recombination between viral genotypes rhinovirus C45 and C11 leading to RV-C45-cpz1-2013, the strain identified in the Kanyawara chimpanzee community, Uganda, 2013. Analyses were performed in RDP4 (*17*) on aligned rhinovirus C genome sequences of 36 known genotypes. Each alignment entry encoded the full or nearly full polyprotein gene sequence, but some sequences were missing fragments (<400 bp) of their respective 5′-UTRs (Δ seq, yellow box at left). The 3′ poly(A) tail was not included. A recombination event between the 2 viruses shown (GenBank nos. JN837686 and EU840952) is the most likely event among all full alignment comparisons (window size 20 bps) according to 6 of the 9 RDP4 algorithms. The average p values were RDP 2.8 × 10^−81^, GENECONV 3.0 × 10^−70^, MaxChi 1.4 × 10^−18^, Chimaera 2.1 × 10^−21^, SiScan 3.6 × 10^–34^, and 3Seq 1.5 × 10^−27^. BootScan, PhylPro, and LARD made no call for these particular parents. Purple box in the 5′-UTR denotes the 99% breakpoint confidence level (combined). Dashed box indicates the position of the most likely swapped fragment. The (Monte Carlo corrected) probability for this event is 2.8 × 10^−81^. The virus map is scaled to the alignment. Conf, confidence level; RV-C, rhinovirus C; Δ seq, missing sequence; UTR, untranslated region.

**Figure 3:**
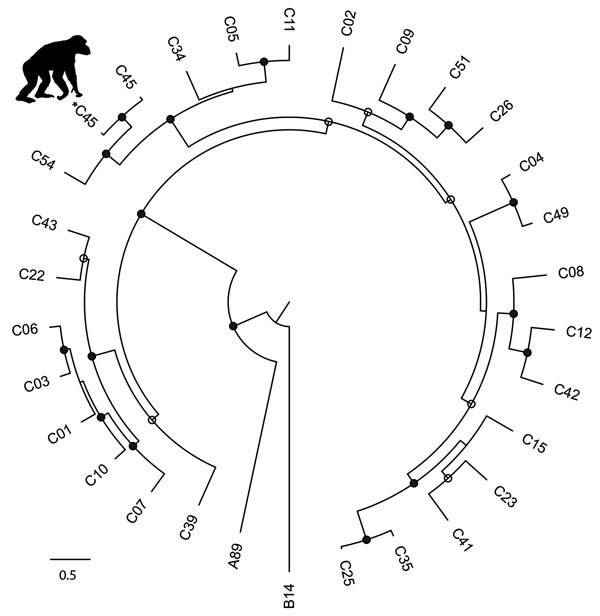
Phylogenetic tree of rhinovirus C variants. The tree was constructed from a codon-based alignment (6,234 positions) of the new chimpanzee-derived sequence identified in the Kanyawara chimpanzee community, Uganda, 2013 (indicated by the asterisk and chimpanzee silhouette), and all human-derived RV-C complete polyprotein gene sequences available in GenBank as of December 18, 2016, with rhinoviruses A and B from the RefSeq database included as outgroups. We created alignments using the MAFFT algorithm (*18*) implemented in the computer program Translator X (*19*), with the Gblocks algorithm (*20*) applied to remove poorly aligned regions. We constructed trees using the maximum-likelihood method implemented in PhyML (*22*), with best-fit models of molecular evolution estimated from the data by using jModelTest (*21*). Circles on nodes indicate statistical confidence on the basis of 1,000 bootstrap replicates of the data (closed circles 100%; open circles >75%). Scale bar indicates nucleotide substitutions per site. GenBank accession numbers and other details of the RV-C sequences included in the analysis are in Technical Appendix Table).

A postmortem analysis was conducted on the infant that died in March (peak of the early phase); other animals that died were not recovered in time for such analyses. The postmortem analysis revealed ecchymotic hemorrhages on the lung surfaces, consolidation of the parenchyma of both lungs (approximately two thirds of both lungs affected), mucoid exudate in the bronchi, hepatomegaly, and hepatic congestion. On the basis of these findings, the cause of death was concluded to be severe acute pneumonia. Deep sequencing of swab samples from this chimpanzee’s oropharynx, trachea, and lung yielded 10,722,035 sequence reads, of which 8,918 had high similarity to rhinovirus C. No other pathogens were detected. Subsequent qPCR confirmed infection with RV-C, with viral loads of 7.41 × 10^6^ copies/swab in the oropharynx, 1.05 × 10^7^ copies/swab in the trachea, and 1.74 × 10^6^ copies/swab in the lung; these values are comparable to the average viral load (7.76 × 10^6^ copies/mL) found in rhinovirus C‒infected children with acute wheezing illness treated in an emergency department (*27*). Further sequencing yielded a near-complete rhinovirus C genome consisting of a complete polyprotein gene of 6,450 bases, a complete 3′ untranslated region (UTR) of 35 bases excluding the poly(A) tail, and a partial 5′-UTR of 480 bases. The viral polyprotein sequence was 94.59% similar at the nucleic acid level and 99.99% similar at the amino acid level to its closest known relative, a rhinovirus C45 sequence (GenBank accession no. JN837686) from a 2-month-old male patient who had a history of mild nasal congestion and discharge in the United States in 2000. RDP4 analyses showed the chimpanzee-derived sequence to be a rhinovirus C45-C11 recombinant, with a breakpoint in the 5′-UTR (Figure 2). In phylogenetic analyses, the virus clustered within known rhinovirus C genotypes, appearing as a sister taxon to the reference rhinovirus C45 isolate (Figure 3). The chimpanzee-derived strain was designated RV-C45-cpz1-2013 to indicate its presumptive serotype and discovery in a chimpanzee in 2013 (GenBank accession no. KY624849).

We confirmed that all swab samples from the deceased infant chimpanzee were positive for rhinovirus C by Luminex assay. Two of the 41 fecal samples collected during the epidemic were positive for enteroviruses, but subsequent molecular typing using published methods (*28*) demonstrated these to be nonrhinovirus enteroviruses, which occur in wild apes without known clinical significance (*14*,*29*,*30*). Fifteen samples were also positive for adenoviruses by Luminex assay. Subsequent typing of these viruses by PCR and direct sequencing of a portion of the hexon gene according to published methods (*31*) was successful for 11 samples. The resulting sequences (GenBank accession nos. KY624838‒KY624848) were identical or closely related to adenoviruses found in fecal samples from apparently healthy wild chimpanzees and other nonhuman primates (*32*–*35*) (Technical Appendix Figure 2).

Genotyping of chimpanzee DNA from fecal samples showed all 41 animals to be homozygous for the *CDHR3*-Y_529_ allele, which is associated with increased susceptibility to rhinovirus C and wheezing illness in humans (*7*). Similarly, all 24 chimpanzee genomes from the Great Ape Genome Project (*25*) were homozygous for the *CDHR3*-Y_529_ allele.

Epidemiologic modeling of the 2013 chimpanzee respiratory epidemic yielded daily transmission rate estimates of 0.85, 0.44, and 0.62 (average 0.68) and duration of infection estimates of 1.6 days, 5.8 days, and 2.1 days (average 3.2 days) for the 3 phases of the epidemic. Together, these parameters equate to basic reproductive numbers (*R*_0_ values) of 1.38, 2.56, and 1.56 for the 3 phases of the epidemic, with an overall *R*_0_ estimate of 1.83 (Technical Appendix Figure 1).

## Discussion

Rhinovirus C is unusual among rhinoviruses because of its clinical severity, unique biochemistry of receptor attachment, and role in modern human evolution (*9*). The analyses we present show that rhinovirus C is also distinguished by its ability to cross primate species barriers and cause severe disease. Although experiments conducted in the 1960s showed rhinoviruses A and B to be capable of infecting chimpanzees in captivity, infections were mild and self-limiting (*36*,*37*). Among other enteroviruses, only poliovirus has been implicated as a cause of death in wild chimpanzees (*38*). By showing that rhinovirus C is highly pathogenic in wild chimpanzees, our findings expand the known host range and clinical consequences of one of the most common causes of human respiratory disease.

Variant RV-C45-cpz1-2013 occupies an unremarkable phylogenetic position among rhinovirus C genomic variants from humans around the world (Figure 3). Although this virus is a recombinant between 2 genotypes, C11 and C45, it is unlikely that this property is related to its origin in a chimpanzee host. Indeed, rhinoviruses often recombine; the parent strains of RV-C45-cpz1-2013 and the genomic location of the recombination event are similar to those described previously (*3*). RV-C45-cpz1-2013 also shares with other rhinovirus C variants the capacity to infect the lower respiratory tract and cause severe disease. Viral load values from this animal’s oropharynx (7.41 × 10^6^ copies/swab), trachea (1.05 × 10^7^ copies/swab), and lung (1.74 × 10^6^ copies/swab) are comparable to the average viral load (7.76 × 10^6^ copies/mL) found in rhinovirus C‒infected children with acute wheezing illness treated in an emergency department (*27*). Although published estimates of rhinovirus epidemiologic transmission parameters are sparse, the values estimated for the 2013 chimpanzee outbreak (transmission rate 0.68/d; duration of infection 3.2 days) are close to those of common cold viruses in humans (transmission rate 0.74/d; duration of infection 3.0 days) (*39*). Despite this similarity, these values are likely similar for many acute respiratory viruses.

Epidemics of respiratory disease have been recorded in ape populations across Africa, but the causes have often remained undiagnosed. For example, during 47 years of wild chimpanzee observation in Gombe, Tanzania, respiratory disease accounted for 48% of illness-related deaths and was the predominant cause of lethal illness in this population (*40*). When noninvasive diagnoses were attempted in other populations, human paramyxoviruses were sometimes implicated (*11*–*14*). However, rhinovirus C could have been missed in outbreaks for which the cause was never identified. Standard molecular diagnostics for rhinoviruses might have failed to detect rhinovirus C, which is genetically divergent from rhinoviruses A and B (*28*). Furthermore, rhinoviruses are adapted to the respiratory tract and are acid sensitive, such that virions dissociate below pH 6.0 (*41*). Because of this property, rhinoviruses are unlikely to survive gastrointestinal transit, and their nucleic acids are unlikely to be found in feces, except during acute and fulminant infections (*42*). Noninvasive diagnostics of ape respiratory disease outbreaks might, therefore, be inherently limited in detecting rhinoviruses, which is unfortunate given that the remote locations in which these endangered apes live often preclude invasive diagnostics (*12*).

In humans, the *CDHR3*-Y_529_ allele is associated with increased susceptibility to rhinovirus C infection (*7*). This elevated risk results from increased cell surface expression of CDHR3, enhanced viral binding to the cellular receptor and increased progeny yields (*6*,*7*). GenBank searches of all published nonhuman primate genome sequences suggest that modern humans are the only primate species in which the *CDHR3*-C_529_ nonrisk allele occurs (A.C. Palmenberg, unpub. data). In agreement with this finding, all chimpanzees genotyped from the Kanyawara community and across Africa were homozygous for the *CDHR3*-Y_529_ risk allele. The fact that only modern humans (and not Neanderthals or Denisovans) possess the nonrisk allele implies that resistance to rhinovirus C infection was selected for only in modern humans (*9*). These results highlight a species-wide susceptibility to rhinovirus C infection among chimpanzees.

Our inference about rhinovirus C as the likely cause of the epidemic is based on clinical samples from a single animal early in the outbreak. We consider it unlikely, however, that rhinovirus C was an incidental finding. The virus was found in this animal’s lung, which was a site of severe pathology, at clinically relevant titers, and paramyxoviruses and other respiratory pathogens were not identified in these samples or in fecal samples from the 41 other animals. All adenoviruses sequenced from fecal samples were similar or identical to adenoviruses that have been documented in apparently healthy wild chimpanzees and other primates (*32*,*34*,*35*,*43*). Finally, clinical signs during the chimpanzee outbreak bore a striking resemblance to those caused by rhinovirus C in susceptible humans, and epidemiologic transmission parameter estimates were consistent with the common cold in human populations (*39*). Nevertheless, we cannot rule out adenoviruses or other undetected co-infecting agents as contributing factors because such infections can enhance the clinical severity of rhinovirus C (*1*). In this regard, we note that simian immunodeficiency virus, which can cause immunodeficiency in wild chimpanzees (*44*), was not detected and is thought to be absent from this chimpanzee population (*45*).

Rhinovirus C is genetically diverse and common among the human populations of sub-Saharan Africa (*9*). Kibale National Park, wherein the home range of the Kanyawara community lies, is frequented by researchers, tourists, and persons living at the periphery of the park, and chimpanzees sometimes leave the park to raid crops in local villages. Accordingly, myriad pathways exist by which the Kanyawara chimpanzees could have been exposed to rhinovirus C from humans. Current guidelines for visiting wild apes in Uganda and other countries are based in part on generalized risks for respiratory disease transmission from humans (e.g., quarantine periods for arriving travelers are mandated at Kanyawara) (*46*). We suggest that specific consideration of rhinovirus C and its particular biologic attributes might improve such guidelines. For example, rhinovirus virions are nonenveloped and might therefore persist in the environment for extended periods of time, even maintaining infectivity after desiccation (*47*).

Long-term records from Kanyawara indicate that respiratory disease outbreaks of varying severity have occurred approximately 2 times per year for at least the past decade, with fatalities occurring every ≈2 years, typically among young animals <5 years of age and adults >35 years of age (R.W. Wrangham, unpub. data). Observations also suggest that respiratory disease outbreaks have occurred in other chimpanzee communities in Kibale at the same times as outbreaks in the Kanyawara community. Kibale contains ≈1,500 chimpanzees in ≈10–20 interconnected communities; that such a population could sustain rhinovirus C or other similar infectious agents in cycles of within-group and between-group transmission is possible, at least for limited time periods (*39*). If respiratory viruses of human origin can circulate independently in wild chimpanzee populations of sufficient size, this fact would be troublesome not only for chimpanzee conservation but also for human health, in that chimpanzees could also serve as a reservoir for human infections.

Although the mandate that researchers and tourists visiting wild apes wear facemasks is controversial, this practice has successfully reduced the transmission of rhinoviruses and other co-infecting agents that exacerbate rhinovirus clinical severity in hospital settings (*48*). Similarly, proper hygiene and the use of hand sanitizer effectively reduce the amounts of rhinovirus on human fingers (*49*). Decades ago, captive chimpanzees were used as study subjects in biomedical research intended to evaluate putative rhinovirus prevention and treatment options (*36*,*37*,*50*). Our results suggest that these investigations could ultimately benefit the wild relatives of the chimpanzees that were the original subjects of these laboratory experimentations. We advocate building upon existing data to engineer novel interventions and prevention strategies for rhinovirus infections in both humans and wild apes.

Technical Appendix Information on rhinovirus C sequences used in phylogenetic analysis, epidemiologic transmission models for the 3 phases of the respiratory disease epidemic, and maximum-likelihood phylogenetic tree of adenoviruses from chimpanzee fecal samples.
