# Supplementary material for: Lethal Respiratory Disease Associated with Human Rhinovirus C in Wild Chimpanzees, Uganda, 2013
Source: Emerg Infect Dis. 2018 Feb;24(2):267–74. doi: 10.3201/eid2402.170778 (PMC5782908; doi:10.3201/eid2402.170778)
Supplement: Technical Appendix — Information on rhinovirus C sequences used in phylogenetic analysis, epidemiologic transmission models for the 3 phases of the respiratory disease epidemic, and maximum-likelihood phylogenetic tree of adenoviruses from chimpanzee fecal samples. [file 17-0778-Techapp-s1.pdf]

Article DOI: https://doi.org/10.3201/eid2402.170778

Lethal Respiratory Disease Associated with Human Rhinovirus C in Wild Chimpanzees, Uganda, 2013

Technical Appendix

Technical Appendix Table. RV-C sequences used in phylogenetic analysis*

Accession	Virus	Isolate
NC_001617	RV-A89	RV-A89
NC_001490	RV-B14	RV-B14
NC_009996	RV-C04	24
**KY624849**	**RV-C45†**	**RV-C45-cpz1–2013**
JN837686	RV-C45	RV-C45-p1084-s3920–2000
DQ875932	RV-C07	NY-074
EF077279	RV-C01	NAT001
EF077280	RV-C02	NAT045
EF186077	RV-C03	RV-QPM
EF582386	RV-C05	25
EF582387	RV-C06	26
EU840952	RV-C11	CL-170085
GQ223227	RV-C08	N4
GQ223228	RV-C09	N10
GQ323774	RV-C10	QCE
GU219984	RV-C15	W10
HQ123440	RV-C35	CU072
HQ123443	RV-C01	CU184
JF317013	RV-C25	LZ269
JF317014	RV-C15	LZY79
JF317015	RV-C51	LZ508
JF317016	RV-C06	LZ651
JF317017	RV-C12	LZY101
JF907574	RV-C49	RV-C49-p1102-sR889–2008
JN205461	RV-C39	WA823M02
JN798567	RV-C03	RV-C03-p1280-s6359–1999
JN837688	RV-C15	RV-C15-p1259-s2935–1999
JN990702	RV-C06	RV-C06-p1031-sR2724–2009
JQ245968	RV-C02	RV-C02-p1264-s3775–1999
JX074056	RV-C43	RV-C43-p1154-sR1124–2009
JX291115	RV-C51	JAL-1
KF734978	RV-C06	1515-MY-10
KF958310	RV-C02	6331
KF958311	RV-C41	2536
KJ675505	RV-C42	570-MY-10
KJ675506	RV-C23	8713-MY-10
KJ675507	RV-C22	3430-MY-10
KM486097	RV-C34	Mex14
KP282614	RV-C54	C54-D3490
KP890662	RV-C01	7383-MY-10
KP890663	RV-C12	3805-MY-10
KP890664	RV-C26	8097-MY-11
KX348031	RV-C35	18455–35

*RV-C, rhinovirus C.
†Isolate from current manuscript.

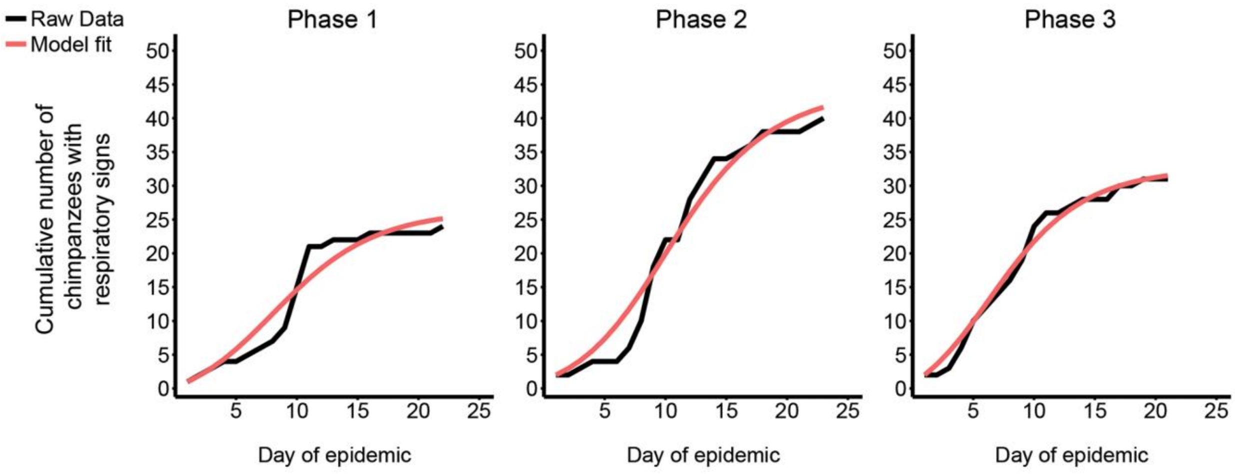

Technical Appendix Figure 1. Epidemiologic transmission model of the 2013 chimpanzee respiratory disease outbreak. We constructed an SIR (susceptible-infectious-removed) model using the following set of ordinary differential equations:

$$\frac{dS}{dt} = \frac{-\beta SI}{N}$$

$$\frac{dI}{dt} = \frac{\beta SI}{N} - \gamma I$$

$$\frac{dR}{dt} = \gamma I$$

where *S* is the number of susceptible chimpanzees, *I* the number of infectious chimpanzees, and *R* the number of recovered chimpanzees; β is the daily transmission probability; γ is the recovery rate; and *N* is the total number of chimpanzees in the population (i.e., *N* = *S* + *I* + *R*). We estimated β and γ and associated CIs by fitting the SIR model to observed cumulative incidence data (*1*) for each phase of the outbreak, and we assumed a total population size of 50 chimpanzees. Here, the basic reproductive number, R_0, is calculated as $R_0 = \frac{\beta}{\gamma}$. Graphs show model predictions superimposed on data from observations of clinical signs for the 3 phases of the epidemic.

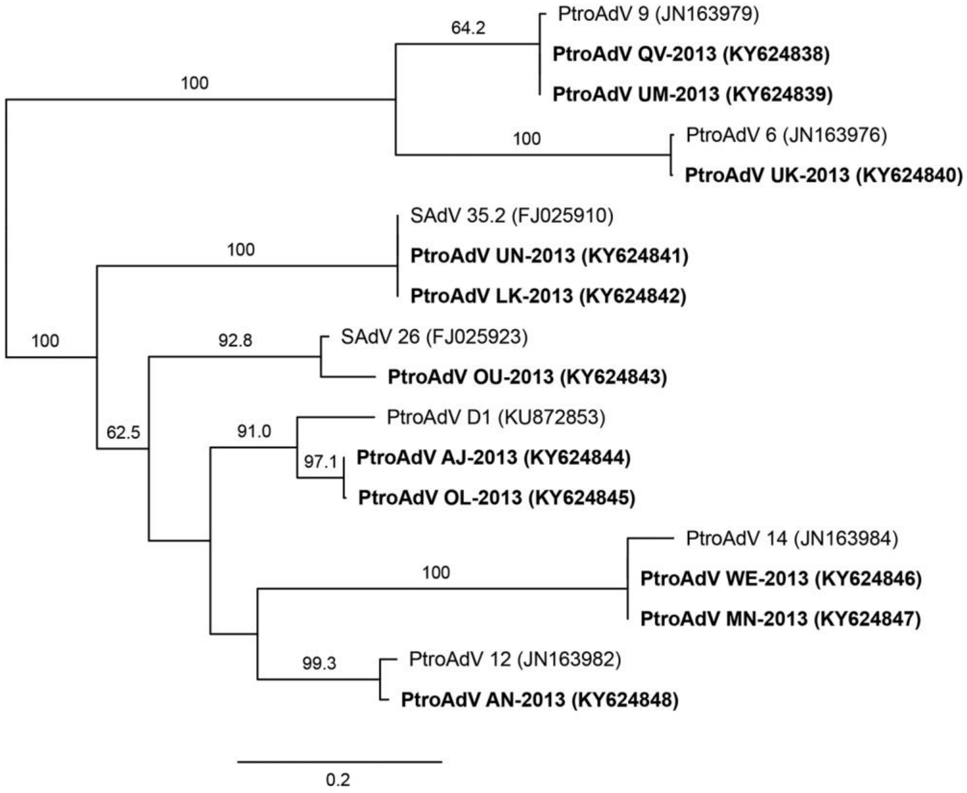

Technical Appendix Figure 2. Maximum-likelihood phylogenetic tree of adenoviruses from fecal samples of wild chimpanzees collected during the respiratory disease epidemic, Uganda, 2013. We constructed the tree from a codon-based alignment (528 positions) of partial adenovirus hexon gene sequences using the same methods as for the tree in Figure 3. Included are sequences generated during this study (bold) and their closest relatives (GenBank accession nos. in parentheses). Numbers above branches indicate statistical confidence on the basis of 1,000 bootstrap replicates (only bootstrap values ≥50% are shown); scale bar indicates nucleotide substitutions per site.

Reference

1. Althaus CL, Low N, Musa EO, Shuaib F, Gsteiger S. Ebola virus disease outbreak in Nigeria: transmission dynamics and rapid control. Epidemics. 2015;11:80–4. PubMed http://dx.doi.org/10.1016/j.epidem.2015.03.001
